# Rapid Brainstem Infiltration of a Cerebellar Glioblastoma

**DOI:** 10.7759/cureus.22643

**Published:** 2022-02-27

**Authors:** Orlando De Jesus, Juan Vigo, María Oliver-Ricart, Juan L Pérez-Berenguer

**Affiliations:** 1 Neurosurgery, University of Puerto Rico, Medical Sciences Campus, San Juan, PRI; 2 Pathology and Laboratory Medicine, University of Puerto Rico, Medical Sciences Campus, San Juan, PRI

**Keywords:** idh mutation, neurosurgery, infiltration, brainstem, glioblastoma, cerebellum

## Abstract

A 79-year-old female complained of a one-month history of imbalance and headache. Brain MRI showed an irregular rim enhancing solid and cystic mass centered in the superomedial left cerebellar hemisphere. Resection of the lesion was recommended; however, the patient opted to undergo the procedure the following month because of the nearby Christmas holidays. When the patient returned 30 days later, a new brain MRI showed an enlargement of the cerebellar mass, extending to the brainstem and infiltrating the left brachium pontis, left posterior aspect of the tegmentum of the pons, and posterolateral medulla oblongata. Subtotal resection was performed without complications, and pathology was compatible with a primary cerebellar glioblastoma negative for IDH1/2 gene mutation. Because of the poor prognosis, the patient and her family members opted for hospice treatment, with the patient dying three weeks later. This case illustrates that cerebellar glioblastoma can rapidly infiltrate the brainstem.

## Introduction

Glioblastoma is an aggressive malignant tumor known to infiltrate adjacent and remote areas. Primary cerebellar glioblastoma is a rare tumor that accounts for just 0.4-3.4% of all cases of glioblastoma [1-3]. Compared with supratentorial glioblastoma, cerebellar glioblastoma occurs predominantly in younger patients, with a mean age of 55 years (range: 19-89) [2-4]. There is a slight male predominance (53-58%) [2-4]. Cerebellar glioblastoma is more frequently associated with neurofibromatosis type I [4]. Primary cerebellar glioblastoma with brainstem invasion occurs in 20-33% of the cases [5-7]. Although it is recognized that a cerebellar glioblastoma can infiltrate the brainstem, how rapidly this can be accomplished has been unreported. We present a case where infiltration occurred in less than 30 days.

## Case presentation

**Figure 1:**
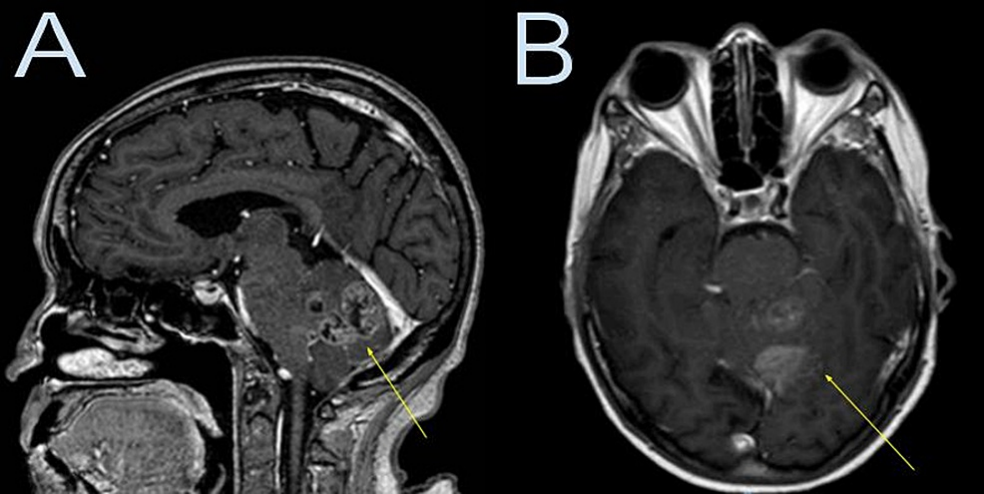
Brain MRI with contrast at the initial evaluation Brain MRI with contrast at the initial evaluation (A: sagittal, B: axial) shows a heterogeneously irregular peripheral rim enhancing solid and cystic, necrotic intra-axial infratentorial mass centered in the superomedial left cerebellar hemisphere (yellow arrow) with extension into the medial aspect of the right cerebellar hemisphere.

A 79-year-old female with a previous right posterior cerebral artery occipital lobe ischemic stroke complained of a one-month history of imbalance and headache. She was on medications for hypothyroidism and hypertension. She denied dizziness, nausea, vomiting, or loss of consciousness. The neurological examination only showed an ataxic gait. There was no papilledema. The brain MRI showed an irregular peripheral rim enhancing solid and cystic intra-axial infratentorial mass centered in the superomedial left cerebellar hemisphere with extension into the medial aspect of the right cerebellar hemisphere measuring 4.3 cm x 4.0 cm x 2.8 cm (Figure 1). The mass showed areas of restricted diffusion towards the periphery. There was no ventricular enlargement. Given its infratentorial location and patient’s age, a metastatic lesion was favored; however, a high-grade primary neoplasm was considered in the differential diagnosis. CT scans of the chest, abdomen, and pelvis were performed to identify any primary malignancy but were negative. At the emergency department, the case was consulted to the neurosurgery service. Biopsy with resection of the mass was recommended; however, the patient stated she would prefer to be discharged and return to the hospital for the procedure the following month because of the upcoming Christmas holidays. The patient was oriented about the risk of not undergoing the procedure this time and was discharged against medical advice. 

**Figure 2:**
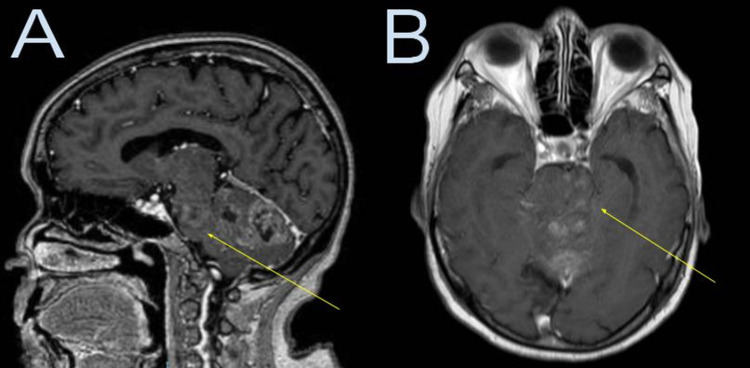
Brain MRI with contrast 30 days later Brain MRI with contrast 30 days later (A: sagittal, B: axial) shows an enlargement of the cerebellar mass, now extending to the brainstem (yellow arrow) with infiltration of the left brachium pontis, the left posterior aspect of the tegmentum of the pons, and posterolateral medulla oblongata.

Thirty days later, the patient returned to the emergency department, where the neurological examination showed no new deficits. Cranial nerve function was intact. Motor and sensory examinations were normal. A new brain MRI showed an enlargement of the cerebellar mass, extending to the brainstem and infiltrating the left brachium pontis, left posterior aspect of the tegmentum of the pons, and posterolateral medulla oblongata (Figure 2). The mass showed restricted diffusion and heterogeneous contrast enhancement areas, with only minimal surrounding vasogenic edema. Based on the new MRI findings, a high-grade glial tumor like a glioblastoma was favored. Surgery via left lateral suboccipital craniotomy was performed without complications with subtotal resection of the tumor. 

**Figure 3:**
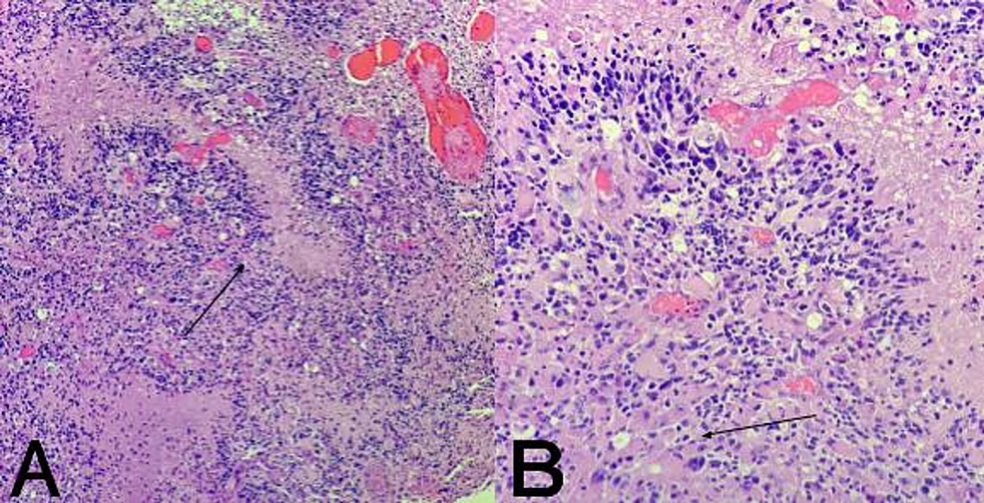
Histopathology of cerebellar glioblastoma IDH-wildtype A: Highly cellular tumor showing pleomorphism with palisading necrosis (black arrow) and vascular proliferation (Hematoxylin and eosin, 100x).  B: Tumor cells show significant nuclear pleomorphism, prominent nucleoli, and a subpopulation of cells with gemistocytic differentiation (black arrow) (Hematoxylin and eosin, 200x). IDH - isocitrate dehydrogenase

**Figure 4:**
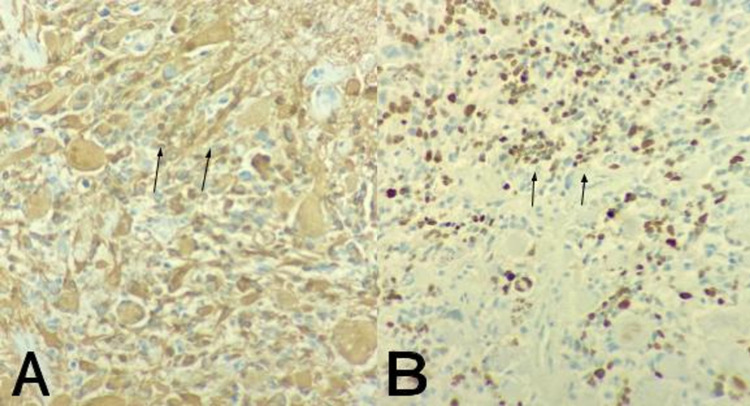
Immunohistochemical profile of cerebellar glioblastoma A: Glial fibrillary acidic protein immunoreactivity diffusely positive in all tumor cells (black arrows) (400x). B: Ki-67 labeling index of 30% (black arrows) (400x).

The histopathology was compatible with a primary cerebellar glioblastoma (isocitrate dehydrogenase (IDH)-wildtype) negative for IDH1/2 gene mutation (Figure 3). The tumor glial fibrillary acidic protein immunoreactivity was diffusely positive in all tumor cells with a Ki-67 labeling index of 30% (Figure 4). Because of the poor prognosis, the patient and her family members opted for hospice treatment, with the patient dying three weeks later. 

## Discussion

Brainstem invasion in primary cerebellar glioblastoma is an adverse prognostic factor and is significantly detrimental to progression-free survival (PFS) and overall survival (OS) [5,6,8]. The presence of leptomeningeal disease in cerebellar glioblastoma results in markedly decreased OS and PFS as well [1]. In a recent study, Picart et al. found that the functional outcomes did not correlate with brainstem infiltration and recommended performing an extensive tumor resection when possible [7]. After total or subtotal resection, the functional outcomes correlated with age and cerebellar hemispheric tumor location [7]. The overall median survival of cerebellar and supratentorial glioblastoma is very similar, approximately eight to nine months [8-12]. However, Picart et al. found that cerebellar glioblastoma was associated with a shorter median overall survival (5.9 vs. 14.2 months) [4].

The histomolecular profile of cerebellar glioblastoma appears to be different from that of supratentorial gliomas. Cerebellar glioblastoma has no IDH1/2 gene mutations but can harbor an H3 K27M mutation in 10-25% of cases [7,13-15]. Hong et al. detected a mutation of IDH1 in two patients with cerebellar glioblastoma who previously had supratentorial anaplastic IDH-mutant gliomas and concluded that some cerebellar glioblastomas are metastasis from supratentorial tumors that spread to the infratentorial area via cerebrospinal fluid pathways or tumor cell migration [16]. Rarely, some patients carry a dual combination of H3 K27M and BRAF V600E mutation, resulting in a better prognosis [16]. Based on these findings, the BRAF V600E mutation could have a more substantial biological significance than the H3 K27M mutation. Reinhardt et al. reclassified their series of cerebellar glioblastomas using array-based methylation analysis and concluded that they represent different molecular brain tumor entities with diverse prognosis and therapeutic options [17]. The new 2021 World Health Organization (WHO) classification of tumors of the central nervous system emphasizes the role of molecular diagnostics in central nervous system tumor classification in addition to other established approaches to tumor characterization, including histology and immunohistochemistry [18]. The new classification recognized new tumor types which can predominate at the cerebellum, such as the high-grade astrocytomas with piloid features [17,18].

This single case with an advanced spread to the brainstem in a short period is not sufficient to predict overall infratentorial glioblastoma aggressiveness. However, it would be interesting if more cases of cerebellar glioblastomas and their growth rate were reported to assess and compare their behavior and draw a more solid conclusion.

## Conclusions

This case demonstrates that cerebellar glioblastoma can rapidly infiltrate the brainstem. Infiltration of the brainstem occurs as the tumor spreads, following along the cerebellopontine fibers. Surgery for cerebellar glioblastoma should be performed without delay to prevent brainstem infiltration. Histopathological diagnosis is essential, including molecular diagnostics to provide the correct prognosis and therapeutic options. Our case confirms that primary cerebellar glioblastoma has no IDH1/2 gene mutations. However, they may harbor several mutations which can alter the prognosis.

## References

[1] Tsung AJ, Prabhu SS, Lei X, Chern JJ, Benjamin Bekele N, Shonka NA (2011). Cerebellar glioblastoma: a retrospective review of 21 patients at a single institution. J Neurooncol.

[2] Cho HJ, Zhao J, Jung SW (2019). Distinct genomic profile and specific targeted drug responses in adult cerebellar glioblastoma. Neuro Oncol.

[3] Adams H, Chaichana KL, Avendaño J, Liu B, Raza SM, Quiñones-Hinojosa A (2013). Adult cerebellar glioblastoma: understanding survival and prognostic factors using a population-based database from 1973 to 2009. World Neurosurg.

[4] Picart T, Barritault M, Berthillier J (2018). Characteristics of cerebellar glioblastomas in adults. J Neurooncol.

[5] Weber DC, Miller RC, Villà S (2006). Outcome and prognostic factors in cerebellar glioblastoma multiforme in adults: a retrospective study from the Rare Cancer Network. Int J Radiat Oncol Biol Phys.

[6] Yang S, Liu J, Wang T, Li X, You C (2013). Cerebellar glioblastoma multiforme: a retrospective study of 28 patients at a single institution. Int J Neurosci.

[7] Picart T, Meyronet D, Pallud J (2021). Management, functional outcomes and survival in a French multicentric series of 118 adult patients with cerebellar glioblastoma. J Cancer Res Clin Oncol.

[8] Gopalakrishnan CV, Dhakoji A, Nair S, Menon G, Neelima R (2012). A retrospective study of primary cerebellar glioblastoma multiforme in adults. J Clin Neurosci.

[9] Babu R, Sharma R, Karikari IO, Owens TR, Friedman AH, Adamson C (2013). Outcome and prognostic factors in adult cerebellar glioblastoma. J Clin Neurosci.

[10] Jeswani S, Nuño M, Folkerts V, Mukherjee D, Black KL, Patil CG (2013). Comparison of survival between cerebellar and supratentorial glioblastoma patients: surveillance, epidemiology, and end results (SEER) analysis. Neurosurgery.

[11] Chandra A, Lopez-Rivera V, Dono A (2021). Comparative analysis of survival outcomes and prognostic factors of supratentorial versus cerebellar glioblastoma in the elderly: does location really matter?. World Neurosurg.

[12] Zhang M, Li R, Pollom EL, Amini A, Dandapani S, Li G (2020). Treatment patterns and outcomes for cerebellar glioblastoma in the concomitant chemoradiation era: a National Cancer database study. J Clin Neurosci.

[13] Tauziède-Espariat A, Saffroy R, Pagès M (2018). Cerebellar high-grade gliomas do not present the same molecular alterations as supratentorial high-grade gliomas and may show histone H3 gene mutations. Clin Neuropathol.

[14] Schreck KC, Ranjan S, Skorupan N, Bettegowda C, Eberhart CG, Ames HM, Holdhoff M (2019). Incidence and clinicopathologic features of H3 K27M mutations in adults with radiographically-determined midline gliomas. J Neurooncol.

[15] Nomura M, Mukasa A, Nagae G (2017). Distinct molecular profile of diffuse cerebellar gliomas. Acta Neuropathol.

[16] Hong B, Banan R, Christians A (2018). Cerebellar glioblastoma: a clinical series with contemporary molecular analysis. Acta Neurochir (Wien).

[17] Reinhardt A, Stichel D, Schrimpf D (2019). Tumors diagnosed as cerebellar glioblastoma comprise distinct molecular entities. Acta Neuropathol Commun.

[18] Louis DN, Perry A, Wesseling P (2021). The 2021 WHO classification of tumors of the central nervous system: a summary. Neuro Oncol.

